# Identification of Drug Resistant *Candida auris*

**DOI:** 10.3389/fmicb.2019.01918

**Published:** 2019-08-20

**Authors:** Milena Kordalewska, David S. Perlin

**Affiliations:** Center for Discovery and Innovation, Hackensack Meridian Health, Nutley, NJ, United States

**Keywords:** *Candida*, *Candida auris*, diagnostics, identification, detection, antifungal drug resistance

## Abstract

*Candida auris* is a multidrug resistant yeast, recognized as a cause of invasive infections and health care associated outbreaks around the world. *C. auris* is of great public health concern, due to its propensity for drug resistance, mode and pace of its transmission, and the possibility that biologic and epidemiologic factors could exacerbate worldwide emergence of *C. auris* infections. Currently, outbreak response is complicated by limited treatment options and inadequate disinfection strategies, as well as by issues (misidentification, long turnaround time) associated with application of commonly used diagnostic tools. Misdiagnosis of *C. auris* is common since many diagnostic platforms available in clinical and public health laboratories depend on reference databases that have not fully incorporated *C. auris*. Moreover, the correlation between minimal inhibitory concentration values (MICs) and clinical outcomes is poorly understood resulting in the absence of *C. auris*-specific breakpoints. New, accurate and fast diagnostic methods have emerged to facilitate effective patient management and improve infection control measures, ultimately reducing the potential for *C. auris* transmission. This review provides an overview of available *C. auris* detection/identification and antifungal susceptibility determination methods and discusses their advantages and limitations. A special emphasis has been placed on culture-independent methods that have recently been developed and offer faster turnaround times.

## Introduction

Over the last few decades, fungal infections are increasingly recognized as a serious concern for human health, especially for immunocompromised patients and those hospitalized with serious underlying diseases. Candidemia and other forms of invasive candidiasis, including infections of normally sterile body fluids, deep tissues, and organs, are the most common nosocomial invasive fungal infections, and are associated with prolonged hospitalization and increased health care costs (Lockhart, 2014; Pfaller et al., 2019). In the US, the most often isolated species is *Candida albicans*, but a trend of increasing number of cases of infection with non-*albicans Candida*, thought to be driven largely by the increasing use of prophylactic antifungal agents such as fluconazole (Deorukhkar et al., 2014), has been reported. Strikingly, non-*albicans Candida* species have been associated with higher mortality and greater antifungal drug resistance than those seen with *C. albicans* infections (Pfaller et al., 2019).

As one of the most prominent emerged pathogens, *Candida auris* has been spreading across the globe causing hospital outbreaks (Meis and Chowdhary, 2018). It is often associated with high level antifungal drug resistance, which limits treatment options. It was first reported in 2009 after being isolated from an external ear canal discharge of a patient in Japan (Satoh et al., 2009). Subsequently, cases of persistent colonization and various types of invasive infections have been reported from many countries located on six continents (Table 1). Bloodstream infection has been the most frequently reported type of invasive infection, with mortality ranging from 30 to 60% (Chowdhary et al., 2017). However, *C. auris* isolates have been recovered from many other types of clinical specimens (Table 1). Currently, *C. auris* is divided into four major clades displaying distinct biological and drug resistance properties (Szekely et al., 2019; Welsh et al., 2019): I, South Asian; II, East Asian; III, South African; IV, South American. *C. auris* infections in other parts of the world, such as the United States and United Kingdom have been caused by strains that are genetically related to these clades (Table 1). Every major clade except for Clade II has been linked to outbreaks with invasive infections. A molecular epidemiological survey performed by Chow et al. in the US showed that travel-related cases, in which patients probably acquired *C. auris* through health-care exposures abroad, are not common (5/73 clinical cases) and that the current epidemic in the United States is dominated by Clade I and Clade IV. Genetic diversity among isolates from the same patients, health-care facilities, and states indicated local and ongoing transmission (however, no apparent transmission even between adjacent states, NY and NJ) (Chow et al., 2018). Several factors, including widespread antifungal use and climate change, have been recently discussed (Jackson et al., 2019), but the exact reasons why this fungus has started spreading in recent years remain unclear.

**TABLE 1:** Published cases of *Candida auris* (as of April 01, 2019).

**Continent**	**Country**	**Clade**	**Isolates source**	**Reliable identification**	**Cases of initial misidentification**	**References**
Asia	China	N/A	Blood, urine, catheter, sputum, bronchoalveolar lavage fluid (BAL)	MALDI-TOF MS, rDNA sequencing	*C. haemulonii* by VITEK 2; *C. famata* by API 20C	Tian et al., 2018; Wang et al., 2018
	India	I	Blood, tissue, pus, BAL, catheter tip, swab (axilla, groin, oral, rectal, skin, vaginal), wound	MALDI-TOF MS, rDNA sequencing	*C. haemulonii, C. famata, C. glabrata* by VITEK 2; *C. sake* by API 20C	Chowdhary et al., 2013; Sarma et al., 2013; Chowdhary et al., 2014; Chatterjee et al., 2015; Kathuria et al., 2015; Kumar et al., 2015; Prakash et al., 2016; Sharma et al., 2016; Lockhart et al., 2017b; Rudramurthy et al., 2017; Chowdhary et al., 2018; Mathur et al., 2018
	Iran	N/A	Ear swab	MALDI-TOF MS, rDNA sequencing	No	Abastabar et al., 2019
	Israel	III	Blood, urine, wound, environment	MALDI-TOF MS and rDNA sequencing	*C. haemulonii* by VITEK 2; *C. parapsilosis* by BD Phoenix	Ben-Ami et al., 2017; Belkin et al., 2018
	Japan	II	Ear discharge	MALDI-TOF MS and rDNA sequencing	*C. haemulonii* by VITEK 2; *Saccharomyces kluyveri* by API ID 32C	Satoh et al., 2009; Iguchi et al., 2018
	Kuwait	N/A	Blood, urine, catheter tip, BAL, pus, endotracheal aspirate, abdominal drain fluid	rDNA sequencing, *C. auris*-specific PCR	*C. haemulonii* by VITEK 2	Emara et al., 2015; Khan et al., 2018a; Khan et al., 2018b; Alobaid and Khan, 2019
	Malaysia	N/A	Blood	rDNA sequencing	*C. haemulonii* by VITEK 2; *Rhodotorula glutinis* by API 20C	Mohd Tap et al., 2018
	Oman	N/A	Blood	MALDI-TOF MS	*C. haemulonii* by API 20C and BD Phoenix	Al-Siyabi et al., 2017; Mohsin et al., 2017
	Pakistan	I	Blood, urine, wound	MALDI-TOF MS	*Saccharomyces cerevisiae* by API 20C	Lockhart et al., 2017b
	Russia	I	Blood, urine, tracheal aspirate	MALDI-TOF MS	No	Barantsevich et al., 2019
	Saudi Arabia	I	Blood, pleural tissue	MALDI-TOF MS	*C. haemulonii* by VITEK 2	Abdalhamid et al., 2018
	Singapore	N/A	Blood, femur tissue	rDNA sequencing	No	Tan and Tan, 2018
	South Korea	II	Blood, catheter tip, pelvic Jackson-Pratt drain, ear discharge	MALDI-TOF MS, rDNA sequencing	*C. haemulonii* by VITEK 2	Lee et al., 2011; Choi et al., 2017; Jung et al., 2019; Kwon et al., 2019a
	Taiwan	N/A	Facial lesion	MALDI-TOF MS, rDNA sequencing	*C. haemulonii* by VITEK 2 and BD Phoenix	Tang et al., 2019
	United Arab Emirates	N/A	Blood	MALDI-TOF MS	*C. haemulonii* by VITEK 2; negative result on BioFire’s Filmarray1 Blood Culture Identification^∗^	Alatoom et al., 2018
Africa	South Africa	III	Blood, cerebrospinal fluid, serous fluid, tissue, urine, respiratory tract specimen, swab (skin, mucosal), catheter tip	MALDI-TOF MS, rDNA sequencing	*C. haemulonii* by VITEK 2; *R. glutinis* by API 20C	Magobo et al., 2014; Prakash et al., 2016; Lockhart et al., 2017b; Govender et al., 2018
South America	Brazil	IV	Blood	MALDI-TOF MS, rDNA sequencing	No	Prakash et al., 2016
	Colombia	IV	Blood, urine, cerebrospinal fluid, peritoneal fluid, ocular secretion, swab (axilla, groin, hands, rectum) healthcare environment	MALDI-TOF MS, rDNA sequencing	*C. haemulonii* by VITEK 2 and BD Phoenix; *C. tropicalis* by MicroScan Walkaway; *C. albicans, C. guilliermondii, C. parapsilosis, C. catenulata, Rhodotorula rubra* by MicroScan; *C. famata* by API Candida	Morales-Lopez et al., 2017; Escandón et al., 2018; Parra-Giraldo et al., 2018; Escandón et al., 2019
	Panama	IV	Blood, urine, catheter tip, pleural fluid	MALDI-TOF MS, rDNA sequencing	*C. haemulonii* by VITEK 2	Arauz et al., 2018
	Venezuela	IV	Blood	rDNA sequencing	*C. haemulonii* by VITEK 2	Calvo et al., 2016; Lockhart et al., 2017b
North America	Canada	N/A	Ear swab	MALDI-TOF MS	No	Schwartz and Hammond, 2017
	United States	I, II, III, IV	Blood, urine, BAL, respiratory tract, sputum, bile fluid, wound, catheter tip, bone, ear, jejunal biopsy, swab (axilla, groin, skin, nares), environment	MALDI-TOF MS, *C. auris-*specific qPCR, rDNA sequencing	Not identified by VITEK MS; *C. haemulonii* by VITEK 2	Azar et al., 2017; Tsay et al., 2017; Vallabhaneni et al., 2017; Chow et al., 2018; Leach et al., 2018; Lesho et al., 2018; Park et al., 2019
Europe	Austria	N/A	Ear swab	MALDI-TOF MS, rDNA sequencing	*C. haemulonii, C. duobushaemulonii* by VITEK 2	Pekard-Amenitsch et al., 2018
	Belgium	N/A	Blood	MALDI-TOF MS, rDNA sequencing	*C. haemulonii* by VITEK 2	Dewaele et al., 2018
	Spain	Close to III	Blood, urine, vascular line, respiratory specimen, catheter tip, swab (rectal), healthcare environment	rDNA sequencing	*C. haemulonii, C. lusitaniae* by VITEK MS; *Saccharomyces cerevisiae* by AuxaColor 2; *C. sake* by API 20C	Ruiz Gaitan et al., 2017, Ruiz-Gaitan A. et al., 2018; Ruiz-Gaitan et al., 2019
	Switzerland	N/A	Tracheal aspirate, groin, auditory canal, urine	MALDI-TOF MS, rDNA sequencing	No	Riat et al., 2018
	United Kingdom	I, II, III	Blood, urine, sputum, cerebrospinal fluid, shunt reservoirs, line, arterial line, femoral line, pleural fluid, cerebral tissue and fluid, catheter tip, swab (groin, skin, oropharynx, wound, pustule), hospital environment	MALDI-TOF MS, rDNA sequencing	No	Borman et al., 2016; Schelenz et al., 2016; Borman et al., 2017; Eyre et al., 2018; Rhodes et al., 2018; Khatamzas et al., 2019
Australia		III^∗∗^	Deep operative sternal bone samples	MALDI-TOF MS and rDNA sequencing	No	Heath et al., 2019

*Countries within continents are listed in an alphabetical order. Clades: I, South Asian; II, East Asian; III, South African; IV, South American. rDNA sequencing: ITS and/or D1/D2 large subunit regions of rDNA sequencing. N/A, information not available. ^∗^test detects only five common Candida species (*C. albicans*, *C. glabrata*, *C. krusei*, *C. parapsilosis*, and *C. tropicalis*). ^∗∗^a man from Kenya visiting Australia*

Major obstacles impacting the control of *C. auris* spread include common misidentification by diagnostic platforms available in clinical and public health laboratories, a poor understanding of resistance to antifungal drugs and disinfectants, and a high propensity to contaminate health care environments which results in transmission of clonal *C. auris* isolates (spread through contact with affected patients and contaminated surfaces). Ultimately, correct detection and identification of the pathogen and its antifungal susceptibility, followed by strict adherence to appropriate treatment and infection prevention and control strategies is crucial for limiting of the spread of *C. auris*.

The aim of this review is to provide an overview of available *C. auris* detection/identification and antifungal susceptibility determination methods and discuss their advantages and limitations. A special emphasis is put on molecular-based and culture-independent methods that have recently been developed and offer faster turnaround times.

## *Candida auris* Identification and Detection in Clinical Samples

There is a vast variety of fungal identification methods used by clinical and public health laboratories all over the world to detect *C. auris*. However, many of them use systems (e.g., VITEK 2, API 20C) for which misidentification with other yeasts has been reported (Table 1). Moreover, because much of the treatment for *Candida* infections is empirical, many institutions do not identify *Candida* to the species level, and if they do it, it is mostly for sterile-site isolates (Lockhart et al., 2017a; Durante et al., 2018). However, the worldwide emergence of *C. auris* heightened public health relevance of the identification of *Candida* to the species level. As broadly discussed by Lockhart et al., species should be determined for isolates recovered from invasive candidiasis cases and selected non-invasive isolates in order to improve detection of *C. auris*. Species identification of isolates from normally sterile body sites enables guidance for initial therapy considering predictable species-specific susceptibility. Moreover, given the emergence of *C. auris*, the Centers for Disease Control and Prevention (CDC) advises species-level identification in the following situations: (1) when clinically indicated in the care of a patient; (2) when a case of *C. auris* infection or colonization has been detected in a facility or unit; (3) when a patient has had an overnight stay in a health care facility in the previous 6 months in a country with *C. auris* transmission. Laboratories should also review past records to identify confirmed or suspected cases, as well as conduct prospective surveillance (Lockhart et al., 2017c).

Accurate identification of *C. auris* infection in the clinical laboratory can be problematic especially when relying on phenotypic characteristics. Institutions without appropriate methodology for *C. auris* species characterization or with isolates that are unidentified or suspect for *C. auris* are strongly advised to contact a reference laboratory for guidance (Durante et al., 2018). In the US, the CDC Advanced Molecular Detection (AMD) program^1^ addresses gaps in technology and workforce knowledge, helps build capacity and provides training at CDC, as well as state and local public health laboratories across the nation.

It is noteworthy, that *C. auris* identification inaccuracies still persist, which has complicated our understanding of the real global prevalence of infections caused by this yeast. Challenges in *C. auris* identification emphasize the importance of local and international collaboration between hospitals (care team), diagnostic laboratories, public health authorities and researchers to optimize diagnostic capacities for rapid identification of emerging pathogens (Lockhart et al., 2017c; Durante et al., 2018).

### Enrichment Culture Protocol for *C. auris* Colonization Screening

The CDC, regional, and state public health laboratories in the United States use Salt Sabouraud Dulcitol enrichment broth procedure for isolation of *C. auris* from clinical skin swabs and environmental sponge surveillance samples. Welsh et al. recommended using dulcitol instead of dextrose as the main carbon source in order to decrease the opportunities of co-incubation of other (non-target) species like *C. glabrata* and *C. parapsilosis* (Welsh et al., 2017).

When screening for *C. auris* colonization, laboratories receive swabs immersed in modified liquid Amies medium. An aliquot (usually 100 μl) of the vortexed swab medium is inoculated into Salt Sabouraud Dulcitol enrichment broth and incubated at 40°C (temperature selective for *C. auris*), with shaking at 250 rpm for at least 5 days. Every sample that becomes turbid is streaked by a loop onto CHROMagar Candida. In a case when a sample remains clear, an aliquot is spread onto CHROMagar Candida. All CHROMagar Candida plates are incubated at 37°C for 48 h and screened for the presence of colony types possible for *C. auris* (Sexton et al., 2018b).

### Phenotypic and Biochemical Methods for *C. auris* Identification

*Candida auris* isolates show smooth white to cream-colored colonies on Sabouraud dextrose agar and appear as beige to pink colonies on CHROMagar Candida medium. However, Bentz et al. (2018) described presence of alternate colors: pink, white, and sectored (dark purple) of smooth and glossy colonies (see photographs^2^), and phenotype switching in isolates recovered from swabs through enrichment broth procedure. By no means recovery of aforementioned colony types on CHROMagar Candida medium can be considered as final identification of *C. auris*, since other yeast, including *C. parapsilosis*, can look alike. Recovered colonies should be further analyzed by one of the reliable identification methods, e.g., rDNA sequencing, *C. auris*-specific PCR/qPCR, or MALDI-TOF MS.

Kumar et al. (2017) proposed using temperature tolerance and CHROMagar supplemented with Pal’s medium in order to morphologically distinguish *C. auris* from *C. haemulonii* and *C. duobushaemulonii*. *C. auris* strains showed confluent growth of white to cream colored smooth colonies at 37°C and 42°C after 24 and 48 h incubation and did not produce pseudohyphae. Contrarily, isolates of the *C. haemulonii* complex showed poor growth of smooth, light-pink colonies at 24 h while at 48 h the growth was semiconfluent with the production of pseudohyphae (Kumar et al., 2017).

*Candida auris* has often been misidentified in conventional diagnostic laboratories using standard biochemical identification systems (Table 1), primarily due to the lack of *C. auris* references in their databases. As databases are being updated, accurate identification becomes more possible, like it happened with VITEK 2 8.01 software version (BioMérieux) in 2018 (Lockhart et al., 2017a; Govender et al., 2018), however misidentifications of strains from certain clades have been reported and all *C. duobushaemulonii* should be forwarded for further identification^2^. A high vigilance still needs to be in place when identification process results in *C. haemulonii, C. duobushaemulonii, C. famata, C. glabrata* from (not updated) VITEK 2 (bioMérieux), *C. famata*, *C. sake*, *Rhodotorula glutinis, Saccharomyces cerevisiae* from API 20C AUX (bioMérieux), *C. haemulonii*, *C. parapsilosis*, *C. catenulata* from BD Phoenix (Becton Dickinson), or *C. albicans*, *C. parapsilosis, C. tropicalis, C. famata, C. lusitaniae, C. guilliermondii, C. catenulata, R. rubra* from MicroScan (Beckman Coulter) (Lockhart et al., 2017a; Mizusawa et al., 2017). Consequently, it might be necessary to confirm such identification result, especially for high fluconazole minimal inhibitory concentration (MIC) or multidrug resistant isolates, by rDNA sequencing, *C. auris*-specific PCR/qPCR or MALDI-TOF MS.

### Identification of *C. auris* by MALDI-TOF MS

The matrix-assisted laser desorption ionization time-of-flight mass spectrometry (MALDI-TOF MS) can be used for rapid and accurate identification of microorganisms. MALDI-TOF MS generates characteristic mass spectra, unique for each microorganism, that can be used as their fingerprints. Since the identification process is based on comparison of spectra acquired for a tested sample and a database of spectra of know species, accurate result is reliant on the presence of the sample organism spectrum in the database. That is why absence of identification or misidentification of fungal species by MALDI-TOF MS is essentially due to absence, mistakes or incomplete reference spectra in the database (Croxatto et al., 2012).

Lack of proper spectra in the databases has resulted in the misidentification of *C. auris* as *C. haemulonii* and *C. albicans*, among others, by MALDI-TOF MS (Kim et al., 2016; Wattal et al., 2017). However, once research-use only (RUO) or manufacturer’s databases are updated with *C. auris* and related species spectra, MALDI-TOF MS identification of *C. auris* to the species level appears to be accurate Table 1 and Cendejas-Bueno et al., 2012; Girard et al., 2016; Bao et al., 2018; Vatanshenassan et al., 2019). It is crucial that laboratories review the presence of aforementioned spectra in the database and confirm the laboratory detection capacity by testing a panel of reference strains, e.g., “*Candida auris* Panel” offered by CDC & FDA Antibiotic Resistance (AR) Isolate Bank^3^, which contains *C. auris* isolates from all clades and other yeast species that are related to *C. auris* or are commonly misidentified as *C. auris*.

Evaluated the viability of *C. auris* after various MALDI-TOF MS extraction protocols (on-plate extraction, quick tube extraction, and extended tube extraction) to determine if isolates processing may expose laboratory workers to infectious agents. *C. auris* was effectively killed in all three methods. Additionally, the authors recommended use of the quick tube extraction method since it gave confidence scores not significantly different from the extended one but better than the on-plate method (Sterkel et al., 2018).

In general, compared with conventional identification methods, MALDI-TOF MS has been shown to confer a significant gain of both technician working time (pre-analytical procedure to prepare samples) and turnaround time (automated analytical procedure to obtain results). MALDI-TOF MS is suitable for high-throughput and rapid microbial identification at low costs and is an alternative for conventional laboratory biochemical and molecular identification systems. Even though the running costs are significantly cheaper than those of conventional identification methods (Croxatto et al., 2012), the amount of money required to purchase a MALDI-TOF MS instrument itself is often an obstacle for laboratories, ultimately preventing implementation of the technique.

In summary, the use of MALDI-TOF MS for the identification of clinically relevant yeasts, including *C. auris*, is rapid and accurate providing that the database is constructed with a comprehensive collection of accurately identified reference strains. Currently, accurate identification of *C. auris* can be achieved by using freely available MicrobeNet database validated by CDC experts^4^ or the following MALDI-TOF MS systems: Bruker Biotyper with FDA-approved MALDI Biotyper CA System library (Version Claim 4) or their “research use only” libraries (Versions 2014 [5627] and more recent) and VITEK (MALDI-TOF) MS RUO (with Saramis Ver 4.14 database and Saccharomycetaceae update).

### Molecular-Based Methods for *C. auris* Identification

Molecular techniques have greatly enhanced the diagnosis of causal agents of infectious disease, particularly when the causal agent is difficult to culture and identify. Sequencing of genetic loci and PCR/qPCR assays have successfully been applied for identification of *C. auris* (Table 1). Especially real-time PCR assays bear incomparable potential of being the most efficient tool for high-throughput screening of surveillance samples. If properly validated, they can deliver the diagnostic result within several hours, since the DNA can be isolated directly from the patient specimen without the need of obtaining a colony.

#### Sequencing of Genetic Loci

Sequencing of rDNA genetic loci, namely internal transcribed spacer and D1/D2 region of large subunit (LSU), that are amplified with standard primers, provides accurate identification of *C. auris* and differentiation from other yeast (Table 1). However, as mentioned by Lockhart et al. sequencing is not routinely used to determine isolate species, since the cost, technical demands, and lengthy turnaround times for those without in-house sequencing capacity can make this technique unsuitable for some laboratories (Lockhart et al., 2017a).

It is also worth to mention, that comparative analysis of rDNA sequences should not be used to define relations between strains due to its insufficient discriminatory power, as revised elsewhere (Jeffery-Smith et al., 2018).

#### Nucleic Acid-Based Identification Strategies

Various molecular-based assays, from conventional PCR, through real-time PCR, to more complex T2 magnetic resonance or Loop-mediated Isothermal Amplification (LAMP), have been developed, that can be applied based on the available resources (Table 2). Most of the assays have the ability to provide rapid results - within several hours from the clinical sample delivery. In comparison, other methods, including biochemical automated systems and MALDI-TOF MS, require culture, that can take anywhere from 4 to 14 days.

**TABLE 2:** Molecular-based methods for identification and detection of *Candida auris*.

**Paper**	**Purpose**	**Identification from**	**DNA extraction**	**Assay type**	**Assay specificity**	**Detection limit**
Kordalewska et al., 2017	New assay	Colonies	Quick boiling method (Brillowska-Dabrowska et al., 2010)	PCR and qPCR (SYBR Green)	(1)*C. auris* (PCR and qPCR) (2)*C. auris*, *C. duobushaemulonii*, *C. haemulonii*, and *C. lusitaniae* (qPCR)	(1) 10 CFU/rxn (2) 1000 CFU/rxn
Sexton et al., 2018b	Validation of the assay	Clinical swabs	Bead beating and DNeasy PowerLyzer Microbial Kit (Qiagen)	qPCR (SYBR Green) from Kordalewska et al., 2017	*C. auris*	4 CFU/rxn
Leach et al., 2018	New assay	Clinical swabs, environmental sponges	Bead beating	qPCR (TaqMan)	*C. auris*	1 CFU/rxn
Ahmad et al., 2019	Validation of rapid extraction method	Clinical swabs	MagNA Pure 96 (Roche)	qPCR (TaqMan) from Leach et al., 2018	*C. auris*	1 CFU/10 μl
Theill et al., 2018	New assay	Colonies	N/A	Duplex PCR	*C. auris* and *C. haemulonii*	N/A
Arastehfar et al., 2018	New assay	Colonies	CTAB method (Gupta et al., 2004)	Tetraplex PCR	*C. auris, C. haemulonii*, *C. duobushaemulonii*, and *C. pseudohaemulonii*,	N/A
Ruiz-Gaitan A.C. et al., 2018	New assay	Colonies	Boiling in 20 mM NaOH	Duplex PCR	*C. auris*	N/A
Khan et al., 2018a	New assay	Colonies	Rapid method using Chelex-100 (Asadzadeh et al., 2015)	PCR	*C. auris*	N/A
Martinez-Murcia et al., 2018	Validation of a commercial kit	Colonies	MagNA Lyser with green beads followed by MagNA Pure 96 (Roche); or GPS DNAcol	GPS MONODOSE dtec-qPCR kit	*C. auris*	5 CFU/rxn
Arastehfar et al., 2019a	New assay	Colonies, spiked serum samples	CTAB method (Gupta et al., 2004)	Tetraplex qPCR (SYBR Green/EvaGreen)	*C. auris, C. haemulonii*, *C. duobushaemulonii*, and *C. pseudohaemulonii*,	10 CFU
Arastehfar et al., 2019b	New assay	Colonies	DNeasy Blood & Tissue Kit (Qiagen) with modifications	Multiplex PCR	21 species including *C. auris*	N/A
Sexton et al., 2018a	Validation of a commercial assay	Clinical swabs	T2Dx	T2 Magnetic Resonance (T2MR) system	*C. auris*	5 CFU/ml
Yamamoto et al., 2018	New assay	Colonies, clinical ear swab, mock environmental samples	Boiling in water (colonies) or Kaneka Easy DNA Extraction kit version 2 (KANEKA Co.) (swabs and environmental samples)	Loop mediated isothermal amplification (LAMP)	*C. auris*	20 CFU/rxn

*N/A, information not available. rxn, reaction.*

Most of the developed assays are highly sensitive, with a detection limit of 1–10 *C. auris* CFU/PCR reaction (Table 2). More importantly, the assays are highly specific and no cross-reactivity is observed with all known closely and distantly related yeasts and other pathogens. Currently, at least three assays have been successfully validated on a panel of clinical swabs (Leach et al., 2018; Sexton et al., 2018a, b), which included samples that were negative for *C. auris* DNA but harbored other organisms. No cross-reactivity was observed, further confirming the high specificity of the assays that can be used for direct detection of *C. auris* without a need of prior culture. It is a huge progress from the *status quo* of axilla/groin composite swabs processing (see: 2.1 Enrichment culture protocol for colonization screening). The delay generated by the requirement of culture has been dramatically limiting the opportunity to quickly respond to outbreaks.

Nucleic acid-based identification strategies may have several technical (availability of equipment and separation of sample prep/amplification/analysis areas) and cost (equipment, reagents) limitations depending on the laboratory infrastructure. Another aspect may be considered both a benefit or limitation, depending on the aim of particular study: DNA from both live and dead *C. auris* cells can be detected, which can be a discrepancy with culture, but shouldn’t be considered a false positive result. A positive result from a non-sterile specimen (skin swab, environmental sponge) is an indicator of past or present *C. auris* colonization.

## Detection of *C. auris* Antifungal Drug Resistance

Antifungal susceptibility testing (AFST) for *C. auris* has been performed using various standardized tests, including Clinical and Laboratory Standards Institute (CLSI) and European Committee on Antimicrobial Susceptibility Testing (EUCAST) broth microdilution methods, the *E*-test gradient diffusion method, and the VITEK 2 antifungal susceptibility system. However, analysis of susceptibility testing results for an emerging fungal species like *C. auris* is complicated by the limited understanding of the correlation between MICs and clinical outcomes (no established breakpoints enabling AFST result interpretation). Currently, CDC provides guidance for *C. auris* MIC interpretation, that based on information gathered for related *Candida* species and expert opinion^5^.

Nevertheless, *C. auris* isolates are often characterized by reduced susceptibility to azoles, polyenes, and echinocandins. Results of susceptibility testing performed on Indian isolates revealed their nearly universal resistance to fluconazole. For that reason, *C. auris* was initially suspected to be intrinsically resistant to this azole drug (Chowdhary et al., 2014, 2018; Kathuria et al., 2015; Arendrup et al., 2017). Moreover, reduced susceptibility of *C. auris* to other triazole antifungal drugs, including voriconazole, posaconazole, itraconazole, and isavuconazole was reported (Chowdhary et al., 2014; Arendrup et al., 2017; Ben-Ami et al., 2017; Lockhart et al., 2017b; Morales-Lopez et al., 2017; Vallabhaneni et al., 2017; Khan et al., 2018a; Rhodes et al., 2018; Ruiz-Gaitan et al., 2019). Such situation when reduced susceptibility to azole class drugs is commonly observed is extremely worrying, since azoles are a mainstay in the treatment of *Candida* infections and antifungals other than fluconazole might be unavailable in resource-limited countries. Nowadays, as more susceptibility information has become available for isolates from around the world and fluconazole susceptible isolates (2–8 mg/l MIC) have been reported, it is believed that fluconazole resistance is an acquired phenomenon in *C. auris* (Lockhart et al., 2017a). Furthermore, polyene antifungal drug amphotericin B can show variable activity against *C. auris* isolates (Shin et al., 2012; Sarma et al., 2013; Magobo et al., 2014; Calvo et al., 2016; Prakash et al., 2016; Sharma et al., 2016; Lockhart et al., 2017b; Vallabhaneni et al., 2017; Khan et al., 2018a; Rhodes et al., 2018; Escandón et al., 2019; Ruiz-Gaitan et al., 2019). The concern about resistance to azoles and amphotericin B has led to the recommendation for the use of echinocandins as a first-line therapy (Chowdhary et al., 2016; Public Health England, 2017). However, reports of *C. auris* isolates showing elevated MIC to one or more echinocandins have been published (Chowdhary et al., 2014, 2018; Sharma et al., 2016; Arendrup et al., 2017; Lockhart et al., 2017b; Vallabhaneni et al., 2017; Kordalewska et al., 2018; Rhodes et al., 2018). Strikingly, some *C. auris* isolates have demonstrated reduced susceptibility to multiple classes of antifungal agents, raising the possibility of pan-drug resistance (Kathuria et al., 2015; Lockhart et al., 2017b).

### Broth Microdilution Methods

As already mentioned, at present, there are no antifungal clinical breakpoints reported for *C. auris* by CLSI or EUCAST. Tentative epidemiological cut off values (ECVs) were proposed in a recent study, where MIC distributions of 123 clinical *C. auris* isolates from India were analyzed (Arendrup et al., 2017). The ECV, which defines the upper limit of the wild type susceptible population of clinical isolates, provides a measure of potential drug resistance. Determined ECVs are valuable in the analysis of MICs of isolates from the South Asian clade, yet their application to isolates from other clades may lead to incorrect estimation of potential drug resistance, since there are strong indications that MIC distributions can vary substantially for *C. auris* isolates from different clades (Szekely et al., 2019; Welsh et al., 2019).

We reported a significant challenge with obtaining an accurate AFST MIC readout for caspofungin since all tested isolates exhibited an Eagle effect (also known as the paradoxical growth effect), with the intensities of the Eagle effect varying among the isolates (Kordalewska et al., 2018). However, echinocandins were effective *in vivo* in the treatment of invasive murine candidiasis caused by *C. auris* isolates, despite the presence of the Eagle effect *in vitro* and only determinant impacting the pharmacodynamic response was the *FKS1* genotype. Therefore, as with many *Candida* species, standardized CLSI susceptibility testing with caspofungin should be viewed cautiously or avoided since an Eagle effect results in an overestimation of the resistant population (Kordalewska et al., 2018).

Nearly all laboratories rely on automated systems for routine AFST. These systems are attractive because they can reduce the laboratory workload relative to manual broth microdilution methods, but they often underperform. Although Kwon et al. observed essential (96.7%) agreement between VITEK 2 and CLSI methods in fluconazole AFST (Kwon et al., 2019b), others have reported its suboptimal performance for amphotericin B (Kathuria et al., 2015), and a cautionary approach is warranted for automated AFST systems.

Therefore, an analysis of susceptibility results obtained by phenotypic tests is not always straightforward and may have difficulties defining susceptible or resistant isolates. More knowledge about clade-specific susceptibility patterns (MICs distribution) is crucial to support the global effort to fight *C. auris* infections efficiently.

## MALDI-TOF

The MALDI Biotyper antibiotic susceptibility test-rapid assay (MBT ASTRA) for rapid AFST. *C. auris* cells were inoculated into RPMI 1640 with 2-fold serial dilutions of echinocandins (anidulafungin, micafungin, and caspofungin) and incubated at 37°C with shaking for 6 h. Later the cells were washed, lysed and deposited in a grid together with matrix, and spectra were acquired and analyzed. A comparison between MBT ASTRA and the CLSI broth microdilution assay results detected a sensitivity and specificity of 100% and 98% for anidulafungin, and 100 and 95.5% for micafungin, respectively. A categorical agreement of 98% and 96% was calculated for the two methods. For caspofungin, sensitivity and specificity of 100 and 73% were found, respectively, with a categorical agreement of 82%. MBT ASTRA has a potential to detect echinocandin non-susceptible *C. auris* isolates within 6 h, which makes it a promising candidate for AFST in clinical laboratories in the future (Vatanshenassan et al., 2019).

### Molecular-Based Methods

Availability of methods for rapid and accurate identification of *C. auris* antifungal drug resistance is of urgent need. Molecular-based tests have the potential to become part of the clinical laboratory routine for resistance detection to help direct therapy and enhance epidemiological surveillance. Since azole and echinocandin resistance in *C. auris* are closely associated with specific *ERG11* and *FKS1* mutations, respectively (Healey et al., 2018; Kordalewska et al., 2018), their analysis can deliver a valuable information on antifungal drug resistance, and facilitate implementation of effective antifungal therapy. Regretfully, at this time there is no clear explanation for the molecular mechanism of *C. auris* amphotericin B resistance. The role of non-synonymous mutations in a transcription factor similar to *FLO8* or membrane transporter described by Escandón et al. (2019) needs to be further investigated. It is also possible that the mechanism of amphotericin B resistance is not mutation-based but regulated at the transcription level (Munoz et al., 2018).

#### Genetic Basis of *C. auris* Antifungal Drug Resistance

Several groups have sequenced genes encoding targets for azoles (Chowdhary et al., 2018; Healey et al., 2018; Kwon et al., 2019b) and echinocandins (Berkow and Lockhart, 2018; Chowdhary et al., 2018; Kordalewska et al., 2018), while the others performed whole genome sequencing (WGS) (Lockhart et al., 2017b; Rhodes et al., 2018), that led to the discovery of mutations responsible for *C. auris* resistance. Echinocandin resistance is mediated through limited mutations S639P (Berkow and Lockhart, 2018) or S639F (Chowdhary et al., 2018; Kordalewska et al., 2018) in *FKS1*, and azole resistance through F126L, Y132F, and K143R in *ERG11* (Lockhart et al., 2017b; Healey et al., 2018; Rhodes et al., 2018). To date, these are the only mutations associated with clinical failures due to azole and echinocandin drugs.

It is crucial to remember that not all mutations detected in target genes confer resistance, and some mutations e.g., the ones leading to K177R, N335S, and E343D amino acid substitutions in Erg11, likely represent genetically evolved clade differences, and do not contribute to any decrease in drug susceptibility (Healey et al., 2018). Any newly identified allele should be carefully investigated to resolve its potential involvement in drug resistance. Validation of resistance properties can be done through molecular engineering approaches (creating/repairing mutation in an organism of the same species, cloning a mutated allele into a model organism) and pharmacodynamic studies in animals.

Moreover, as discussed earlier (see section “Sequencing of Genetic Loci”), and even more accurate for WGS, several factors related to the cost, turnaround time, technology and expertise make sequencing impractical for the regular workflow of clinical microbiology laboratories (Lockhart et al., 2017a).

#### Real-Time PCR Assay for Identification of Mutations in *C. auris ERG11* and *FKS1* Genes

Recently, highly accurate real-time PCR diagnostic platform was developed for rapid identification of mutations in *C. auris ERG11* and *FKS1* genes, conferring azole and echinocandin resistance, respectively. Analysis of melting profiles of molecular beacons, small stem-loop-structured DNA oligonucleotides, was used in conjunction with asymmetric PCR. Since a single-stranded amplicon is generated during asymmetric PCR, the molecular beacon can anneal and generate fluorescence at low temperature. Later, the temperature is increased, and the probe slowly dissociates from the target, which results in the fluorescence intensity decrease. Melting curve analysis (a plot of the fluorescence intensity changes as a function of temperature) enables determination of temperature at which the molecular beacon-target DNA hybrids melt apart (*T*_*m*_). The *T*_*m*_ value is different in a situation when the target and the probe sequences match perfectly and when they are mismatched, thereby providing an ideal tool for typing single-nucleotide polymorphisms e.g., wild-type (WT)/non-WT discrimination (Zhao et al., 2016).

A duplex *ERG11* assay and a simplex *FKS1* HS1 assay were developed to identify the most prominent resistance-associated mutations (Y132F and K143R in *ERG11*; S639F in *FKS1* HS1) within 2 h. The molecular diagnostic results from the assays were 100% concordant with DNA sequencing results. Advantages of the assays include easy interpretation of the results since the testing strain can be easily identified by comparing the *T*_*m*_ value with those generated by reference strains representing WT and mutant target region; multiplex potential by combining several probes; and expandable readiness for novel/unreported mutation detection, because the assay will be able to pick up a novel mutation by simply incorporating the corresponding genotype into the library without the need for assay redesign (Hou et al., 2019).

This platform may potentially be used for direct detection of *C. auris* antifungal drug resistance markers in clinical swabs, dramatically reducing the time needed to obtain information on isolate susceptibility. Thus, it has the potential to overcome the deficiencies of existing *in vitro* susceptibility-based assays to identify azole- and/or echinocandin-resistant *C. auris* and holds promise as a surrogate diagnostic method to direct antifungal therapy more effectively.

## Conclusion

Efforts to control *C. auris* have posed multiple challenges that include developing and evaluating new diagnostics, understanding its epidemiology, and identifying means to minimize transmission among patients. With emergence of less common fungal opportunists, newly described species, and species that can exhibit antifungal resistance mechanisms, one must take care to not blindly accept results from commonly available methods. New molecular methods used in tandem with classical methods give the diagnostician a broad and ever-increasing armamentarium at their disposal to provide the clinician with the most reliable information to ensure the best possible patient care and outcome.

## Author Contributions

MK contributed to the conceptualization and writing the original draft of the manuscript. DP contributed to the reviewing and editing of the manuscript.

## Conflict of Interest Statement

DP receives funding from the U.S. National Institutes of Health and contracts with The Centers for Disease Control and Prevention, Amplyx, Astellas, Cidara, and Scynexis. He serves on advisory boards for Amplyx, Astellas, Cidara, Matinas, N8 Medical and Scynexis. In addition, DP has an issued U.S. patent concerning echinocandin resistance. The remaining author declares that the research was conducted in the absence of any commercial or financial relationships that could be construed as a potential conflict of interest.

## Footnotes

1 www.cdc.gov/amd

2 www.cdc.gov/fungal/candida-auris/recommendations.html

3 www.cdc.gov/drugresistance/resistance-bank

4 www.cdc.gov/microbenet

5 www.cdc.gov/fungal/candida-auris/c-auris-antifungal.html

## References

[B1] Abastabar M. Haghani I. Ahangarkani F. Rezai M. S. Taghizadeh Armaki M. Roodgari S. (2019). *Candida auris* otomycosis in Iran and review of recent literature. *Mycoses* 62 101–105. 10.1111/myc.12886 30585653

[B2] Abdalhamid B. Almaghrabi R. Althawadi S. Omrani A. (2018). First report of *Candida auris* infections from Saudi Arabia. *J. Infect. Public Health* 11 598–599. 10.1016/j.jiph.2018.05.010 29895475

[B3] Ahmad A. Spencer J. E. Lockhart S. R. Singleton S. Petway D. J. Bagarozzi D. A. Jr. (2019). A high-throughput and rapid method for accurate identification of emerging multidrug-resistant *Candida auris*. *Mycoses* 62 513–518. 10.1111/myc.12907 30801778 PMC10888143

[B4] Alatoom A. Sartawi M. Lawlor K. AbdelWareth L. Thomsen J. Nusair A. (2018). Persistent candidemia despite appropriate fungal therapy: first case of *Candida auris* from the United Arab Emirates. *Int. J. Infect. Dis.* 70 36–37. 10.1016/j.ijid.2018.02.005 29452247

[B5] Alobaid K. Khan Z. (2019). Epidemiologic characteristics of adult candidemic patients in a secondary hospital in Kuwait: a retrospective study. *J. Mycol. Med.* 29 35–38. 10.1016/j.mycmed.2018.12.001 30578148

[B6] Al-Siyabi T. Al Busaidi I. Balkhair A. Al-Muharrmi Z. Al-Salti M. Al’Adawi B. (2017). First report of *Candida auris* in Oman: clinical and microbiological description of five candidemia cases. *J. Infect.* 75 373–376. 10.1016/j.jinf.2017.05.016 28579303

[B7] Arastehfar A. Fang W. Badali H. Vaezi A. Jiang W. Liao W. (2018). Low-cost tetraplex PCR for the global spreading multi-drug resistant fungus, *Candida auris* and its phylogenetic relatives. *Front. Microbiol.* 9:1119. 10.3389/fmicb.2018.01119 29896181 PMC5987591

[B8] Arastehfar A. Fang W. Daneshnia F. Al-Hatmi A. M. Liao W. Pan W. (2019a). Novel multiplex real-time quantitative PCR detecting system approach for direct detection of *Candida auris* and its relatives in spiked serum samples. *Future Microbiol.* 14 33–45. 10.2217/fmb-2018-0227 30539665

[B9] Arastehfar A. Fang W. Pan W. Lackner M. Liao W. Badiee P. (2019b). YEAST PANEL multiplex PCR for identification of clinically important yeast species: stepwise diagnostic strategy, useful for developing countries. *Diagn. Microbiol. Infect. Dis.* 93 112–119. 10.1016/j.diagmicrobio.2018.09.007 30377018

[B10] Arauz A. B. Caceres D. H. Santiago E. Armstrong P. Arosemena S. Ramos C. (2018). Isolation of *Candida auris* from 9 patients in Central America: importance of accurate diagnosis and susceptibility testing. *Mycoses* 61 44–47. 10.1111/myc.12709 28945325

[B11] Arendrup M. C. Prakash A. Meletiadis J. Sharma C. Chowdhary A. (2017). Comparison of EUCAST and CLSI reference microdilution MICs of eight antifungal compounds for *Candida auris* and associated tentative epidemiological cutoff values. *Antimicrob. Agents Chemother.* 61:e00485-17. 10.1128/AAC.00485-17 28416539 PMC5444165

[B12] Asadzadeh M. Ahmad S. Hagen F. Meis J. F. Al-Sweih N. Khan Z. (2015). Simple, low-cost detection of *Candida parapsilosis* complex isolates and molecular fingerprinting of *Candida orthopsilosis* strains in Kuwait by ITS region sequencing and amplified fragment length polymorphism analysis. *PLoS One* 10:e0142880. 10.1371/journal.pone.0142880 26580965 PMC4651534

[B13] Azar M. M. Turbett S. E. Fishman J. A. Pierce V. M. (2017). Donor-derived transmission of *Candida auris* during lung transplantation. *Clin. Infect. Dis.* 65 1040–1042. 10.1093/cid/cix460 28520901

[B14] Bao J. R. Master R. N. Azad K. N. Schwab D. A. Clark R. B. Jones R. S. (2018). Rapid, accurate identification of *Candida auris* by using a novel matrix-assisted laser desorption ionization-time of flight mass spectrometry (MALDI-TOF MS) database (library). *J. Clin. Microbiol.* 56:e01700-17. 10.1128/JCM.01700-17 PMC5869814 29367296

[B15] Barantsevich N. E. Orlova O. E. Shlyakhto E. V. Johnson E. M. Woodford N. Lass-Floerl C. (2019). Emergence of *Candida auris* in Russia. *J. Hosp. Infect.* 102 445–448. 10.1016/j.jhin.2019.02.021 30851375

[B16] Belkin A. Gazit Z. Keller N. Ben-Ami R. Wieder-Finesod A. Novikov A. (2018). *Candida auris* infection leading to nosocomial transmission, Israel, 2017. *Emerg. Infect. Dis.* 24 801–804. 10.3201/eid2404.171715 29553329 PMC5875262

[B17] Ben-Ami R. Berman J. Novikov A. Bash E. Shachor-Meyouhas Y. Zakin S. (2017). Multidrug-resistant *Candida haemulonii* and *C. auris*, Tel Aviv, Israel. *Emerg. Infect. Dis.* 23 195–203. 10.3201/eid2302.161486 28098529 PMC5324804

[B18] Bentz M. L. Sexton D. J. Welsh R. M. Litvintseva A. P. (2018). Phenotypic switching in newly emerged multidrug-resistant pathogen *Candida auris*. *Med. Mycol.* 10.1093/mmy/myy100 [Epub ahead of print]. 30329075

[B19] Berkow E. L. Lockhart S. R. (2018). Activity of CD101, a long-acting echinocandin, against clinical isolates of *Candida auris*. *Diagn. Microbiol. Infect. Dis.* 90 196–197. 10.1016/j.diagmicrobio.2017.10.021 29307565

[B20] Borman A. M. Szekely A. Johnson E. M. (2016). Comparative pathogenicity of united kingdom isolates of the emerging pathogen *Candida auris* and other key pathogenic *Candida* species. *mSphere* 1:e00189-16. 10.1128/mSphere.00189-16 27547827 PMC4990711

[B21] Borman A. M. Szekely A. Johnson E. M. (2017). Isolates of the emerging pathogen *Candida auris* present in the UK have several geographic origins. *Med. Mycol.* 55 563–567. 10.1093/mmy/myw147 28204557

[B22] Brillowska-Dabrowska A. Nielsen S. S. Nielsen H. V. Arendrup M. C. (2010). Optimized 5-hour multiplex PCR test for the detection of tinea unguium: performance in a routine PCR laboratory. *Med. Mycol.* 48 828–831. 10.3109/13693780903531579 20105101

[B23] Calvo B. Melo A. S. Perozo-Mena A. Hernandez M. Francisco E. C. Hagen F. (2016). First report of *Candida auris* in America: clinical and microbiological aspects of 18 episodes of candidemia. *J. Infect.* 73 369–374. 10.1016/j.jinf.2016.07.008 27452195

[B24] Cendejas-Bueno E. Kolecka A. Alastruey-Izquierdo A. Theelen B. Groenewald M. Kostrzewa M. (2012). Reclassification of the *Candida haemulonii* complex as *Candida haemulonii* (*C. haemulonii* group I), *C. duobushaemulonii* sp. nov. (*C. haemulonii* group II), and *C. haemulonii* var. *vulnera* var. nov.: three multiresistant human pathogenic yeasts. *J. Clin. Microbiol.* 50 3641–3651. 10.1128/JCM.02248-12 22952266 PMC3486233

[B25] Chatterjee S. Alampalli S. V. Nageshan R. K. Chettiar S. T. Joshi S. Tatu U. S. (2015). Draft genome of a commonly misdiagnosed multidrug resistant pathogen *Candida auris*. *BMC Genomics* 16:686. 10.1186/s12864-015-1863-z 26346253 PMC4562351

[B26] Choi H. I. An J. Hwang J. J. Moon S. Y. Son J. S. (2017). Otomastoiditis caused by *Candida auris*: case report and literature review. *Mycoses* 60 488–492. 10.1111/myc.12617 28378904

[B27] Chow N. A. Gade L. Tsay S. V. Forsberg K. Greenko J. A. Southwick K. L. (2018). Multiple introductions and subsequent transmission of multidrug-resistant *Candida auris* in the USA: a molecular epidemiological survey. *Lancet Infect. Dis.* 18 1377–1384. 10.1016/S1473-3099(18)30597-8 30293877 PMC6556114

[B28] Chowdhary A. Anil Kumar V. Sharma C. Prakash A. Agarwal K. Babu R. (2014). Multidrug-resistant endemic clonal strain of *Candida auris* in India. *Eur. J. Clin. Microbiol. Infect. Dis.* 33 919–926. 10.1007/s10096-013-2027-1 24357342

[B29] Chowdhary A. Prakash A. Sharma C. Kordalewska M. Kumar A. Sarma S. (2018). A multicentre study of antifungal susceptibility patterns among 350 *Candida auris* isolates (2009-17) in India: role of the ERG11 and FKS1 genes in azole and echinocandin resistance. *J. Antimicrob. Chemother.* 73 891–899. 10.1093/jac/dkx480 29325167

[B30] Chowdhary A. Sharma C. Duggal S. Agarwal K. Prakash A. Singh P. K. (2013). New clonal strain of *Candida auris*, Delhi, India. *Emerg. Infect. Dis.* 19 1670–1673. 10.3201/eid1910.130393 24048006 PMC3810747

[B31] Chowdhary A. Sharma C. Meis J. F. (2017). *Candida auris*: a rapidly emerging cause of hospital-acquired multidrug-resistant fungal infections globally. *PLoS Pathog.* 13:e1006290. 10.1371/journal.ppat.1006290 28542486 PMC5436850

[B32] Chowdhary A. Voss A. Meis J. F. (2016). Multidrug-resistant *Candida auris*: ‘new kid on the block’ in hospital-associated infections? *J. Hosp. Infect.* 94 209–212. 10.1016/j.jhin.2016.08.004 27634564

[B33] Croxatto A. Prod’hom G. Greub G. (2012). Applications of MALDI-TOF mass spectrometry in clinical diagnostic microbiology. *FEMS Microbiol. Rev.* 36 380–407. 10.1111/j.1574-6976.2011.00298.x 22092265

[B34] Deorukhkar S. C. Saini S. Mathew S. (2014). Non-*albicans Candida* infection: an emerging threat. *Interdiscip. Perspect. Infect. Dis.* 2014:615958. 10.1155/2014/615958 25404942 PMC4227454

[B35] Dewaele K. Frans J. Smismans A. Ho E. Tollens T. Lagrou K. (2018). First case of *Candida auris* infection in Belgium in a surgical patient from Kuwait. *Acta Clin. Belg.* 10.1080/17843286.2018.1555114 [Epub ahead of print]. 30514182

[B36] Durante A. J. Maloney M. H. Leung V. H. Razeq J. H. Banach D. B. (2018). Challenges in identifying *Candida auris* in hospital clinical laboratories: a need for hospital and public health laboratory collaboration in rapid identification of an emerging pathogen. *Infect. Control Hosp. Epidemiol.* 39 1015–1016. 10.1017/ice.2018.133 29966540

[B37] Emara M. Ahmad S. Khan Z. Joseph L. Al-Obaid I. Purohit P. (2015). *Candida auris* candidemia in Kuwait, 2014. *Emerg. Infect. Dis.* 21 1091–1092. 10.3201/eid2106.150270 25989098 PMC4451886

[B38] Escandón P. Caceres D. H. Espinosa-Bode A. Rivera S. Armstrong P. Vallabhaneni S. (2018). Notes from the field: surveillance for *Candida auris* - Colombia, September 2016-May 2017. *MMWR Morb. Mortal. Wkly. Rep.* 67 459–460. 10.15585/mmwr.mm6715a6 29672473 PMC6191104

[B39] Escandón P. Chow N. A. Caceres D. H. Gade L. Berkow E. L. Armstrong P. (2019). Molecular epidemiology of *Candida auris* in Colombia reveals a highly related, countrywide colonization with regional patterns in amphotericin B resistance. *Clin. Infect. Dis.* 68 15–21. 10.1093/cid/ciy411 29788045

[B40] Eyre D. W. Sheppard A. E. Madder H. Moir I. Moroney R. Quan T. P. (2018). A *Candida auris* outbreak and its control in an intensive care setting. *N. Engl. J. Med.* 379 1322–1331. 10.1056/NEJMoa1714373 30281988

[B41] Girard V. Mailler S. Chetry M. Vidal C. Durand G. van Belkum A. (2016). Identification and typing of the emerging pathogen *Candida auris* by matrix-assisted laser desorption ionisation time of flight mass spectrometry. *Mycoses* 59 535–538. 10.1111/myc.12519 27292939

[B42] Govender N. P. Magobo R. E. Mpembe R. Mhlanga M. Matlapeng P. Corcoran C. (2018). *Candida auris* in South Africa, 2012-2016. *Emerg. Infect. Dis.* 24 2036–2040. 10.3201/eid2411.180368 30334713 PMC6200016

[B43] Gupta A. K. Boekhout T. Theelen B. Summerbell R. Batra R. (2004). Identification and typing of *Malassezia* species by amplified fragment length polymorphism and sequence analyses of the internal transcribed spacer and large-subunit regions of ribosomal DNA. *J. Clin. Microbiol.* 42 4253–4260. 10.1128/JCM.42.9.4253-4260.2004 15365020 PMC516278

[B44] Healey K. R. Kordalewska M. Jimenez Ortigosa C. Singh A. Berrio I. Chowdhary A. (2018). Limited ERG11 mutations identified in isolates of *Candida auris* directly contribute to reduced azole susceptibility. *Antimicrob. Agents Chemother.* 62:e01427-18. 10.1128/AAC.01427-18 30082281 PMC6153782

[B45] Heath C. H. Dyer J. R. Pang S. Coombs G. W. Gardam D. J. (2019). *Candida auris* sternal osteomyelitis in a man from Kenya visiting Australia, 2015. *Emerg. Infect. Dis.* 25 192–194. 10.3201/eid2501.181321 30561310 PMC6302580

[B46] Hou X. Lee A. Jimenez-Ortigosa C. Kordalewska M. Perlin D. S. Zhao Y. (2019). Rapid detection of *ERG11*-associated azole resistance and *FKS*-associated echinocandin resistance in *Candida auris*. *Antimicrob. Agents Chemother.* 63:e01811-18. 10.1128/AAC.01811-18 30397051 PMC6325222

[B47] Iguchi S. Mizushima R. Kamada K. Itakura Y. Yoshida A. Uzawa Y. (2018). The second *Candida auris* isolate from aural discharge in Japan. *Jpn. J. Infect. Dis.* 71 174–175. 10.7883/yoken.JJID.2017.466 29491246

[B48] Jackson B. R. Chow N. Forsberg K. Litvintseva A. P. Lockhart S. R. Welsh R. (2019). On the origins of a species: what might explain the rise of *Candida auris*? *J. Fungi* 5:58. 10.3390/jof5030058 31284576 PMC6787658

[B49] Jeffery-Smith A. Taori S. K. Schelenz S. Jeffery K. Johnson E. M. Borman A. (2018). *Candida auris*: a review of the literature. *Clin. Microbiol. Rev.* 31:e00029-17. 10.1128/CMR.00029-17 29142078 PMC5740969

[B50] Jung J. Kim M. J. Kim J. Y. Lee J. Y. Kwak S. H. Hong M. J. (2019). *Candida auris* colonization or infection of the ear: a single-center study in South Korea from 2016 to 2018. *Med. Mycol.* 10.1093/mmy/myz020 [Epub ahead of print]. 30874806

[B51] Kathuria S. Singh P. K. Sharma C. Prakash A. Masih A. Kumar A. (2015). Multidrug-resistant *Candida auris* misidentified as *Candida haemulonii*: characterization by matrix-assisted laser desorption ionization-time of flight mass spectrometry and DNA sequencing and its antifungal susceptibility profile variability by Vitek 2, CLSI broth microdilution, and Etest method. *J. Clin. Microbiol.* 53 1823–1830. 10.1128/JCM.00367-15 25809970 PMC4432077

[B52] Khan Z. Ahmad S. Al-Sweih N. Joseph L. Alfouzan W. Asadzadeh M. (2018a). Increasing prevalence, molecular characterization and antifungal drug susceptibility of serial *Candida auris* isolates in Kuwait. *PLoS One* 13:e0195743. 10.1371/journal.pone.0195743 29630658 PMC5891028

[B53] Khan Z. Ahmad S. Benwan K. Purohit P. Al-Obaid I. Bafna R. (2018b). Invasive *Candida auris* infections in Kuwait hospitals: epidemiology, antifungal treatment and outcome. *Infection* 46 641–650. 10.1007/s15010-018-1164-y 29949089

[B54] Khatamzas E. Madder H. Jeffery K. (2019). Neurosurgical device-associated infections due to *Candida auris* - Three cases from a single tertiary center. *J. Infect.* 78 409–421. 10.1016/j.jinf.2019.02.004 30742893

[B55] Kim T. H. Kweon O. J. Kim H. R. Lee M. K. (2016). Identification of uncommon *Candida* species using commercial identification systems. *J. Microbiol. Biotechnol.* 26 2206–2213. 10.4014/jmb.1609.09012 27713213

[B56] Kordalewska M. Lee A. Park S. Berrio I. Chowdhary A. Zhao Y. (2018). Understanding echinocandin resistance in the emerging pathogen *Candida auris*. *Antimicrob. Agents Chemother.* 62:e00238-18. 10.1128/AAC.00238-18 29632013 PMC5971591

[B57] Kordalewska M. Zhao Y. Lockhart S. R. Chowdhary A. Berrio I. Perlin D. S. (2017). Rapid and accurate molecular identification of the emerging multidrug-resistant pathogen *Candida auris*. *J. Clin. Microbiol.* 55 2445–2452. 10.1128/JCM.00630-17 28539346 PMC5527423

[B58] Kumar A. Sachu A. Mohan K. Vinod V. Dinesh K. Karim S. (2017). Simple low cost differentiation of *Candida auris* from *Candida haemulonii* complex using CHROMagar Candida medium supplemented with Pal’s medium. *Rev. Iberoam. Micol.* 34 109–111. 10.1016/j.riam.2016.11.004 28392225

[B59] Kumar D. Banerjee T. Pratap C. B. Tilak R. (2015). Itraconazole-resistant *Candida auris* with phospholipase, proteinase and hemolysin activity from a case of vulvovaginitis. *J. Infect. Dev. Ctries.* 9 435–437. 10.3855/jidc.4582 25881537

[B60] Kwon Y. J. Shin J. H. Byeon S. A. Choi M. J. Won E. J. Lee D. (2019a). *Candida auris* clinical isolates from South Korea: identification, antifungal susceptibility, and genotyping. *J. Clin. Microbiol.* 57:e01624-18. 10.1128/JCM.01624-18 PMC6440790 30728190

[B61] Kwon Y. J. Shin J. H. Byun S. A. Choi M. J. Won E. J. Lee D. (2019b). *Candida auris* clinical isolates from South Korea: identification, antifungal susceptibility, and genotyping. *J. Clin. Microbiol.* 57:e01624-18. 10.1128/JCM.01624-18 PMC6440790 30728190

[B62] Leach L. Zhu Y. Chaturvedi S. (2018). Development and validation of a real-time PCR assay for rapid detection of *Candida auris* from surveillance samples. *J. Clin. Microbiol.* 56:e01223-17. 10.1128/JCM.01223-17 29187562 PMC5786737

[B63] Lee W. G. Shin J. H. Uh Y. Kang M. G. Kim S. H. Park K. H. (2011). First three reported cases of nosocomial fungemia caused by *Candida auris*. *J. Clin. Microbiol.* 49 3139–3142. 10.1128/JCM.00319-11 21715586 PMC3165631

[B64] Lesho E. P. Bronstein M. Z. McGann P. Stam J. Kwak Y. Maybank R. (2018). Importation, mitigation, and genomic epidemiology of *Candida auris* at a large teaching hospital. *Infect. Control Hosp. Epidemiol.* 39 53–57. 10.1017/ice.2017.231 29208056

[B65] Lockhart S. R. (2014). Current epidemiology of *Candida* infection. *Clin. Microbiol. Newsl.* 36 131–136. 10.1016/j.clinmicnews.2014.08.001 PMC11951269 40161481

[B66] Lockhart S. R. Berkow E. L. Chow N. Welsh R. M. (2017a). *Candida auris* for the clinical microbiology laboratory: not your grandfather’s *Candida* species. *Clin. Microbiol. Newsl.* 39 99–103. 10.1016/j.clinmicnews.2017.06.003 29503491 PMC5831145

[B67] Lockhart S. R. Etienne K. A. Vallabhaneni S. Farooqi J. Chowdhary A. Govender N. P. (2017b). Simultaneous emergence of multidrug-resistant *Candida auris* on 3 continents confirmed by whole-genome sequencing and epidemiological analyses. *Clin. Infect. Dis.* 64 134–140. 10.1093/cid/ciw691 27988485 PMC5215215

[B68] Lockhart S. R. Jackson B. R. Vallabhaneni S. Ostrosky-Zeichner L. Pappas P. G. Chiller T. (2017c). Thinking beyond the common *Candida* species: need for species-level identification of *Candida* due to the emergence of multidrug-resistant *Candida auris*. *J. Clin. Microbiol.* 55 3324–3327. 10.1128/JCM.01355-17 28904185 PMC5703798

[B69] Magobo R. E. Corcoran C. Seetharam S. Govender N. P. (2014). *Candida auris*-associated candidemia, South Africa. *Emerg. Infect. Dis.* 20 1250–1251. 10.3201/eid2007.131765 24963796 PMC4073876

[B70] Martinez-Murcia A. Navarro A. Bru G. Chowdhary A. Hagen F. Meis J. F. (2018). Internal validation of GPS() MONODOSE CanAur dtec-qPCR kit following the UNE/EN ISO/IEC 17025:2005 for detection of the emerging yeast *Candida auris*. *Mycoses* 61 877–884. 10.1111/myc.12834 30059175

[B71] Mathur P. Hasan F. Singh P. K. Malhotra R. Walia K. Chowdhary A. (2018). Five-year profile of candidaemia at an Indian trauma centre: high rates of *Candida auris* blood stream infections. *Mycoses* 61 674–680. 10.1111/myc.12790 29738604

[B72] Meis J. F. Chowdhary A. (2018). *Candida auris*: a global fungal public health threat. *Lancet Infect. Dis.* 18 1298–1299. 10.1016/s1473-3099(18)30609-1 30293876

[B73] Mizusawa M. Miller H. Green R. Lee R. Durante M. Perkins R. (2017). Can multidrug-resistant *Candida auris* be reliably identified in clinical microbiology laboratories? *J. Clin. Microbiol.* 55 638–640. 10.1128/jcm.02202-16 27881617 PMC5277535

[B74] Mohd Tap R. Lim T. C. Kamarudin N. A. Ginsapu S. J. Abd Razak M. F. Ahmad N. (2018). A fatal case of *Candida auris* and *Candida tropicalis* candidemia in neutropenic patient. *Mycopathologia* 183 559–564. 10.1007/s11046-018-0244-y 29383574 PMC5958168

[B75] Mohsin J. Hagen F. Al-Balushi Z. A. M. de Hoog G. S. Chowdhary A. Meis J. F. (2017). The first cases of *Candida auris* candidaemia in Oman. *Mycoses* 60 569–575. 10.1111/myc.12647 28685887

[B76] Morales-Lopez S. E. Parra-Giraldo C. M. Ceballos-Garzon A. Martinez H. P. Rodriguez G. J. Alvarez-Moreno C. A. (2017). Invasive infections with multidrug-resistant yeast *Candida auris*, Colombia. *Emerg. Infect. Dis.* 23 162–164. 10.3201/eid2301.161497 27983941 PMC5176232

[B77] Munoz J. F. Gade L. Chow N. A. Loparev V. N. Juieng P. Berkow E. L. (2018). Genomic insights into multidrug-resistance, mating and virulence in *Candida auris* and related emerging species. *Nat. Commun.* 9:5346. 10.1038/s41467-018-07779-6 30559369 PMC6297351

[B78] Park J. Y. Bradley N. Brooks S. Burney S. Wassner C. (2019). Management of patients with *Candida auris* fungemia at community hospital, Brooklyn, New York, USA, 2016-2018(1). *Emerg. Infect. Dis.* 25 601–602. 10.3201/eid2503.180927 30789336 PMC6390749

[B79] Parra-Giraldo C. M. Valderrama S. L. Cortes-Fraile G. Garzon J. R. Ariza B. E. Morio F. (2018). First report of sporadic cases of *Candida auris* in Colombia. *Int. J. Infect. Dis.* 69 63–67. 10.1016/j.ijid.2018.01.034 29421668

[B80] Pekard-Amenitsch S. Schriebl A. Posawetz W. Willinger B. Kolli B. Buzina W. (2018). Isolation of *Candida auris* from ear of otherwise healthy patient, Austria, 2018. *Emerg. Infect. Dis.* 24 1596–1597. 10.3201/eid2408.180495 30016243 PMC6056136

[B81] Pfaller M. A. Diekema D. J. Turnidge J. D. Castanheira M. Jones R. N. (2019). Twenty years of the SENTRY antifungal surveillance program: results for *Candida* species from 1997-2016. *Open Forum Infect. Dis.* 6(Suppl. 1), S79–S94. 10.1093/ofid/ofy358 30895218 PMC6419901

[B82] Prakash A. Sharma C. Singh A. Kumar Singh P. Kumar A. Hagen F. (2016). Evidence of genotypic diversity among *Candida auris* isolates by multilocus sequence typing, matrix-assisted laser desorption ionization time-of-flight mass spectrometry and amplified fragment length polymorphism. *Clin. Microbiol. Infect.* 22 277.e1–277.e9. 10.1016/j.cmi.2015.10.022 26548511

[B83] Public Health England (2017). *Guidance for the Laboratory Investigation, Management and Infection Prevention and Control for Cases of Candida auris.* London: Public Health England.

[B84] Rhodes J. Abdolrasouli A. Farrer R. A. Cuomo C. A. Aanensen D. M. Armstrong-James D. (2018). Genomic epidemiology of the UK outbreak of the emerging human fungal pathogen *Candida auris*. *Emerg. Microbes Infect.* 7:43. 10.1038/s41426-018-0045-x 29593275 PMC5874254

[B85] Riat A. Neofytos D. Coste A. Harbarth S. Bizzini A. Grandbastien B. (2018). First case of *Candida auris* in Switzerland: discussion about preventive strategies. *Swiss Med. Wkly.* 148:w14622. 10.4414/smw.2018.14622 29698547

[B86] Rudramurthy S. M. Chakrabarti A. Paul R. A. Sood P. Kaur H. Capoor M. R. (2017). *Candida auris* candidaemia in Indian ICUs: analysis of risk factors. *J. Antimicrob. Chemother.* 72 1794–1801. 10.1093/jac/dkx034 28333181

[B87] Ruiz Gaitan A. C. Moret A. Lopez Hontangas J. L. Molina J. M. Aleixandre Lopez A. I. Cabezas A. H. (2017). Nosocomial fungemia by *Candida auris*: first four reported cases in continental Europe. *Rev. Iberoam. Micol.* 34 23–27. 10.1016/j.riam.2016.11.002 28131716

[B88] Ruiz-Gaitan A. Moret A. M. Tasias-Pitarch M. Aleixandre-Lopez A. I. Martinez-Morel H. Calabuig E. (2018). An outbreak due to *Candida auris* with prolonged colonisation and candidaemia in a tertiary care European hospital. *Mycoses* 61 498–505. 10.1111/myc.12781 29655180

[B89] Ruiz-Gaitan A. C. Fernandez-Pereira J. Valentin E. Tormo-Mas M. A. Eraso E. Peman J. (2018). Molecular identification of *Candida auris* by PCR amplification of species-specific GPI protein-encoding genes. *Int. J. Med. Microbiol.* 308 812–818. 10.1016/j.ijmm.2018.06.014 30025998

[B90] Ruiz-Gaitan A. C. Canton E. Fernandez-Rivero M. E. Ramirez P. Peman J. (2019). Outbreak of *Candida auris* in Spain: a comparison of antifungal activity by three methods with published data. *Int. J. Antimicrob. Agents* 53 541–546. 10.1016/j.ijantimicag.2019.02.005 30769198

[B91] Sarma S. Kumar N. Sharma S. Govil D. Ali T. Mehta Y. (2013). Candidemia caused by amphotericin B and fluconazole resistant *Candida auris*. *Indian J. Med. Microbiol.* 31 90–91. 23508441 10.4103/0255-0857.108746

[B92] Satoh K. Makimura K. Hasumi Y. Nishiyama Y. Uchida K. Yamaguchi H. (2009). *Candida auris* sp. nov., a novel ascomycetous yeast isolated from the external ear canal of an inpatient in a Japanese hospital. *Microbiol. Immunol.* 53 41–44. 10.1111/j.1348-0421.2008.00083.x 19161556

[B93] Schelenz S. Hagen F. Rhodes J. L. Abdolrasouli A. Chowdhary A. Hall A. (2016). First hospital outbreak of the globally emerging *Candida auris* in a European hospital. *Antimicrob. Resist. Infect. Control* 5:35. 10.1186/s13756-016-0132-5 27777756 PMC5069812

[B94] Schwartz I. S. Hammond G. W. (2017). First reported case of multidrug-resistant *Candida auris* in Canada. *Can. Commun. Dis. Rep.* 43 150–153. 10.14745/ccdr.v43i78a02 29770082 PMC5764715

[B95] Sexton D. J. Bentz M. L. Welsh R. M. Litvintseva A. P. (2018a). Evaluation of a new T2 Magnetic Resonance assay for rapid detection of emergent fungal pathogen *Candida auris* on clinical skin swab samples. *Mycoses* 61 786–790. 10.1111/myc.12817 29938838

[B96] Sexton D. J. Kordalewska M. Bentz M. L. Welsh R. M. Perlin D. S. Litvintseva A. P. (2018b). Direct detection of emergent fungal pathogen *Candida auris* in clinical skin swabs by SYBR green-based quantitative PCR assay. *J. Clin. Microbiol.* 56:e01337-18. 10.1128/JCM.01337-18 30232130 PMC6258843

[B97] Sharma C. Kumar N. Pandey R. Meis J. F. Chowdhary A. (2016). Whole genome sequencing of emerging multidrug resistant *Candida auris* isolates in India demonstrates low genetic variation. *New Microbes New Infect.* 13 77–82. 10.1016/j.nmni.2016.07.003 27617098 PMC5006800

[B98] Shin J. H. Kim M. N. Jang S. J. Ju M. Y. Kim S. H. Shin M. G. (2012). Detection of amphotericin B resistance in *Candida haemulonii* and closely related species by use of the Etest, Vitek-2 yeast susceptibility system, and CLSI and EUCAST broth microdilution methods. *J. Clin. Microbiol.* 50 1852–1855. 10.1128/JCM.06440-11 22442324 PMC3372122

[B99] Sterkel A. Bateman A. Valley A. Warshauer D. (2018). Viability of *Candida auris* and other *Candida* species after various matrix-assisted laser desorption ionization-time of flight (MALDI-TOF) mass spectrometry-based extraction protocols. *J. Clin. Microbiol.* 56:e00886-18. 10.1128/JCM.00886-18 PMC6113450 29950335

[B100] Szekely A. Borman A. M. Johnson E. M. (2019). *Candida auris* isolates of the Southern Asian and South African lineages exhibit different phenotypic and antifungal susceptibility profiles in vitro. *J. Clin. Microbiol.* 57:e02055-18. 10.1128/JCM.02055-18 30867237 PMC6498034

[B101] Tan Y. E. Tan A. L. (2018). Arrival of *Candida auris* fungus in Singapore: report of the first 3 cases. *Ann. Acad. Med. Singapore* 47 260–262. 30120434

[B102] Tang H. J. Lai C. C. Lai F. J. Li S. Y. Liang H. Y. Hsueh P. R. (2019). Emergence of multidrug-resistant *Candida auris* in Taiwan. *Int. J. Antimicrob. Agents* 53 705–706. 10.1016/j.ijantimicag.2019.02.011 30822471

[B103] Theill L. Dudiuk C. Morales-Lopez S. Berrio I. Rodriguez J. Y. Marin A. (2018). Single-tube classical PCR for *Candida auris* and *Candida haemulonii* identification. *Rev. Iberoam. Micol.* 35 110–112. 10.1016/j.riam.2018.01.003 29685376

[B104] Tian S. Rong C. Nian H. Li F. Chu Y. Cheng S. (2018). First cases and risk factors of super yeast *Candida auris* infection or colonization from Shenyang, China. *Emerg. Microbes Infect.* 7:128. 10.1038/s41426-018-0131-0 29992959 PMC6041263

[B105] Tsay S. Welsh R. M. Adams E. H. Chow N. A. Gade L. Berkow E. L. (2017). Notes from the field: ongoing transmission of *Candida auris* in health care facilities - United States, June 2016-May 2017. *MMWR Morb. Mortal. Wkly. Rep.* 66 514–515. 10.15585/mmwr.mm6619a7 28520710 PMC5657645

[B106] Vallabhaneni S. Kallen A. Tsay S. Chow N. Welsh R. Kerins J. (2017). Investigation of the first seven reported cases of *Candida auris*, a globally emerging invasive, multidrug-resistant fungus-United States, May 2013-August 2016. *Am. J. Transplant.* 17 296–299. 10.1111/ajt.14121 28029734

[B107] Vatanshenassan M. Boekhout T. Meis J. F. Berman J. Chowdhary A. Ben-Ami R. (2019). *Candida auris* identification and rapid antifungal susceptibility testing against echinocandins by MALDI-TOF MS. *Front. Cell. Infect. Microbiol.* 9:20. 10.3389/fcimb.2019.00020 30834236 PMC6387932

[B108] Wang X. Bing J. Zheng Q. Zhang F. Liu J. Yue H. (2018). The first isolate of *Candida auris* in China: clinical and biological aspects. *Emerg. Microbes Infect.* 7:93. 10.1038/s41426-018-0095-0 29777096 PMC5959928

[B109] Wattal C. Oberoi J. K. Goel N. Raveendran R. Khanna S. (2017). Matrix-assisted laser desorption ionization time of flight mass spectrometry (MALDI-TOF MS) for rapid identification of micro-organisms in the routine clinical microbiology laboratory. *Eur. J. Clin. Microbiol. Infect. Dis.* 36 807–812. 10.1007/s10096-016-2864-9 27987046

[B110] Welsh R. M. Bentz M. L. Shams A. Houston H. Lyons A. Rose L. J. (2017). Survival, persistence, and isolation of the emerging multidrug-resistant pathogenic yeast *Candida auris* on a plastic health care surface. *J. Clin. Microbiol.* 55 2996–3005. 10.1128/JCM.00921-17 28747370 PMC5625385

[B111] Welsh R. M. Sexton D. J. Forsberg K. Vallabhaneni S. Litvintseva A. (2019). Insights into the unique nature of the East Asian clade of the emerging pathogenic yeast *Candida auris*. *J. Clin. Microbiol.* 57:e00007-19. 10.1128/JCM.00007-19 30760535 PMC6440783

[B112] Yamamoto M. Alshahni M. M. Tamura T. Satoh K. Iguchi S. Kikuchi K. (2018). Rapid detection of *Candida auris* based on loop-mediated isothermal amplification (LAMP). *J. Clin. Microbiol.* 56:e00591-18. 10.1128/JCM.00591-18 PMC6113467 29950329

[B113] Zhao Y. Nagasaki Y. Kordalewska M. Press E. G. Shields R. K. Nguyen M. H. (2016). Rapid detection of FKS-associated echinocandin resistance in *Candida glabrata*. *Antimicrob. Agents Chemother.* 60 6573–6577. 10.1128/AAC.01574-16 27550360 PMC5075061

